# Targeting neoantigens for cancer immunotherapy

**DOI:** 10.1186/s40364-021-00315-7

**Published:** 2021-07-28

**Authors:** Xuan Zhao, Xiaoxin Pan, Yi Wang, Yi Zhang

**Affiliations:** 1 Biotherapy Center & Cancer Center, The First Affiliated Hospital of Zhengzhou University, 450052 Zhengzhou, China; 2 State Key Laboratory of Esophageal Cancer Prevention & Treatment, Zhengzhou University, 450052 Zhengzhou, China; 3 Shenzhen NeoCura Biotechnology Corporation, 518055 Shenzhen, China; 4 School of Life Sciences, Zhengzhou University, 450052 Zhengzhou, China; 5 Henan Key Laboratory for Tumor Immunology and Biotherapy, 450052 Zhengzhou, China

**Keywords:** Neoantigen vaccine, Cancer immunotherapy, Precision medicine

## Abstract

Neoantigens, a type of tumor-specific antigens derived from non-synonymous mutations, have recently been characterized as attractive targets for cancer immunotherapy. Owing to the development of next-generation sequencing and utilization of machine-learning algorithms, it has become feasible to computationally predict neoantigens by depicting genetic alterations, aberrant post-transcriptional mRNA processing and abnormal mRNA translation events within tumor tissues. Consequently, neoantigen-based therapies such as cancer vaccines have been widely tested in clinical trials and have demonstrated promising safety and efficacy, opening a new era for cancer immunotherapy. We systematically summarize recent advances in the identification of both personalized and public neoantigens, neoantigen formulations and neoantigen-based clinical trials in this review. Moreover, we discuss future techniques and strategies for neoantigen-based cancer treatment either as a monotherapy or as a combination therapy with radiotherapy, chemotherapy or immune checkpoint inhibitors.

## Background

Cancer ranks second among diseases in terms of mortality [1, 2]. Traditional cancer treatment strategies, including surgery, radiotherapy, chemotherapy, hormone therapy, and targeted drugs, focus mainly on either reducing the viability or inhibiting the growth of tumor cells, by directly acting on them. In recent decades, cancer immunotherapy has shown great potential to combat cancer, and the immune system is activated to target malignant tumors [3]. In healthy tissues, immune surveillance guarantees the elimination of somatic mutations; unfortunately, in advanced cancer, immune surveillance fails to do so, resulting in immune escape and tumorigenesis [4, 5]. In principle, cancer immunotherapy aims to elicit and magnify the cytotoxic activity of tumor-cell-targeting immune cells, overcome immunosuppression in the tumor tissues, and boost the host immune system to fight against cancer.

**Fig. 1:**
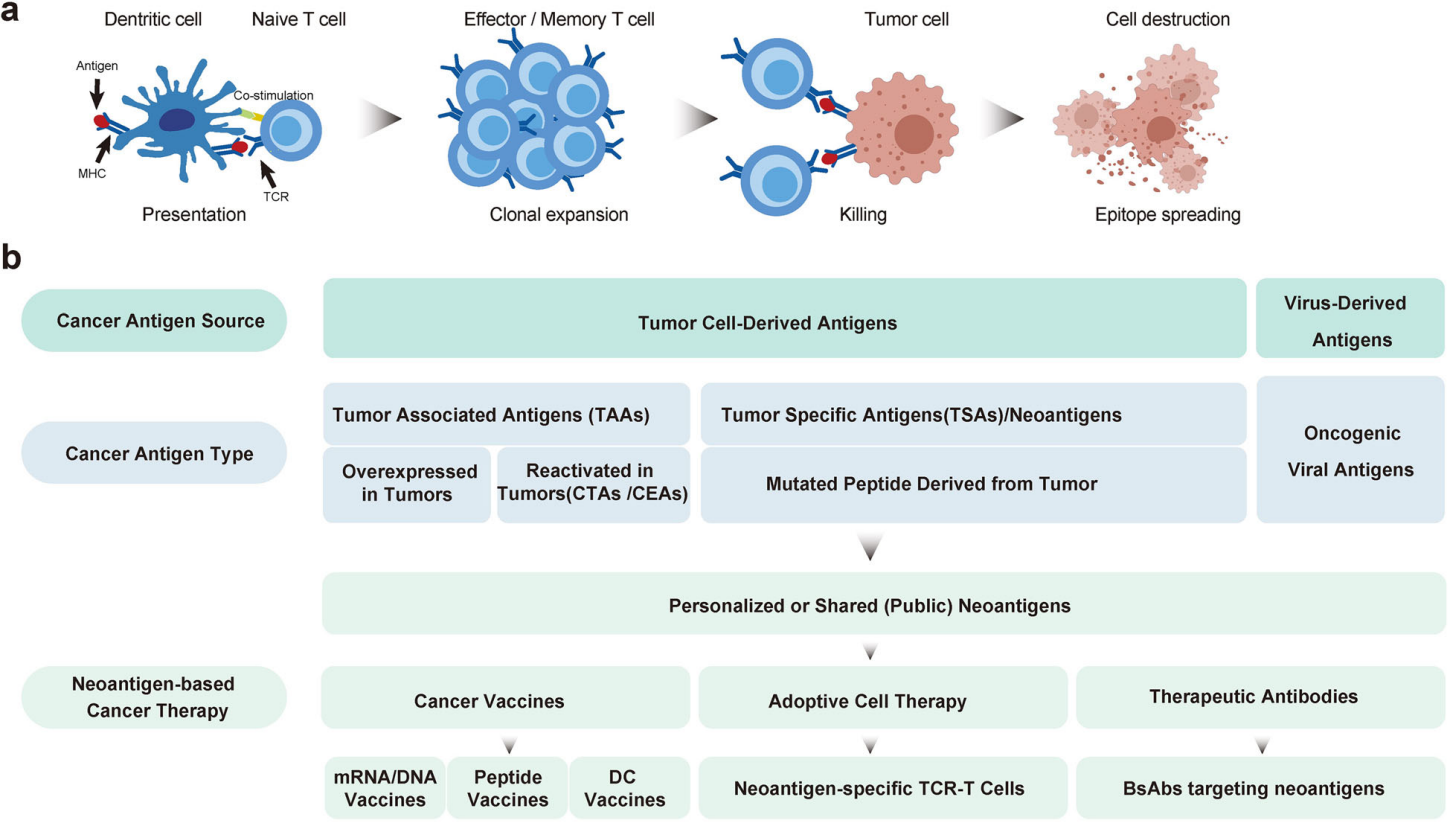
Cancer antigen-specific cell destruction, tumor antigen subtypes, and neoantigen-based cancer therapy.** a** T cell-mediated tumor cell killing via antigen–TCR-MHC interaction **(b)** Cancer antigen source, type and neoantigen-based cancer therapy. Tumor antigens derived from either endogenous tumor-cells or exogenous viruses can be classified into three categories: tumor associated antigens (TAAs), tumor-specific antigens (TSAs)/neoantigens, and oncogenic viral proteins. A fraction of personalized neoantigens comprises public neoantigens that are shared among patients. Neoantigen-based therapy could be categorized into cancer vaccines, adoptive cell therapy, and therapeutic antibodies

Cancer immunotherapy has proven to be clinically effective in multiple types of cancers. Immune checkpoint inhibitors (ICIs; also known as immune checkpoint blockades, ICBs, ICPs, or CPIs) are therapeutic monoclonal antibodies against immune checkpoint molecules, such as programmed cell death protein-1 (PD-1), programmed cell death ligand-1 (PD-L1), and cytotoxic T lymphocyte antigen-4 (CTLA-4) [6]. Inhibition of these key immunosuppressing molecules has noteworthy clinical effects on several types of cancers[7]. Aside from ICI drugs, adoptive cell transfer (ACT) therapy provides an alternative set of immunotherapy treatments. In this approach, functional autogenous immune cells that target human leukocyte antigen (HLA)-antigen complexes are isolated from the tumor tissues of patients. Then, tumor-infiltrating lymphocytes (TILs) are amplified or engineered *in vitro*, and subsequently infused back into the patient to achieve precise cytotoxicity on tumor cells expressing the targeting antigens. ACT therapy involves the use of unmodified TILs, T cells with engineered T-cell receptor (TCR) fragments (TCR-Ts) [8, 9], and chimeric antibody receptor-engineered T cells (CAR-Ts) [10–15]. The clinical outcome of both ICI and ACT treatments depends on the presence of tumor-derived antigens, which are the core of tumor immunotherapy in killing tumor cells by T lymphocytes [16]. Generally, TCRs on the surface of CD4^+^ and CD8^+^ T lymphocytes recognize antigens (Ag) or epitopes displayed by major histocompatibility complex (MHC) I and MHC II molecules, respectively. When MHC–Ag–TCR tertiary complexes are formed as recognition signals with co-stimulatory signals (interaction between CD28-CD80/CD86), they can trigger signal transduction in T cells, and then destruct the target cells (Fig. 1a) [17]. There are three major origin-based categories of tumor antigens arising from different sources: tumor-associated antigens (TAAs), oncogenic virus-derived antigens, and tumor-specific antigens (TSAs, neoantigens) (Fig. 1b). The features of the three types of tumor antigens are summarized in Table 1.

**Table 1:** Tumor antigen summary

Category	Tumor-associated antigens (TAAs)			Oncogenic virus-derived antigens	Tumor-specific antigens (TSAs, neoantigens)	
Expression site	Mostly restricted to tumors, sometimes expressed in normal tissue			Infected cells and virus-related tumor cells	Restricted to tumors	
Origin	Abnormal gene expression			Virus infection	Mutated peptides derived from genomic alterations	
Subset	Overexpressed proteins	Differentiation antigens	Cancer/testis antigens	N/A	Public neoantigens	Private neoantigens
Specificity	Variable	Variable	Good	Ideal	Ideal	
Central tolerance	High	High	Low	None	None	
Prevalence across multiple cancers	High	High	High in certain cancers	High in certain cancers	High	Low

### Tumor-associated antigens

Tumor-associated antigens have several subsets, such as tumor-overexpressed proteins, cancer testicular antigens, and carcinoembryonic antigens. The first generation of immunotherapeutic cancer vaccines was designed to target proteins that are highly expressed in tumor tissues. Most of these proteins are expressed in normal cells, and are significantly upregulated in tumor cells. Examples of TAAs include human epidermal growth factor receptor 2 (HER2/neu, or receptor tyrosine-protein kinase erbB-2, ERBB2), telomerase reverse transcriptase (TERT or hTERT), and tyrosinase (TYR) [18–20].

Cancer testis antigens (CTAs) and cell lineage differentiation antigens (CDAs) are two special subsets of TAAs. CTAs are expressed mainly in germ cells of male adults and are sometimes expressed in the ovary and placenta. [21–23]. New York esophageal squamous cell carcinoma-1 (NY-ESO-1) and melanoma antigen gene (MAGE) superfamily members are the most common CTAs used in adoptive T cell therapy as targets to activate the immune response. A pilot trial conducted by Rosenberg used autologous T lymphocytes engineered with an NY-ESO-1-reactive TCR to treat the metastatic synovial cell sarcoma or melanoma patients with HLA-0201^+^, and these cancers were NY-ESO-1 positive. The results showed overall clinical response rates of 61 % (11/18) for synovial cell sarcomas and 55 % (11/20) for melanoma. It was elucidated that NY-ESO-1, which is often shared by other CTAs, was expressed heterogeneously within tumor cells, such that it may theoretically limit the immunotherapeutic efficacy of genetically engineered T cells against this protein [24].

Similar to CTAs, other tumor-overexpressed TAAs, such as CDAs, prostate-specific antigen (PSA), carcinoembryonic antigens (CEAs), and prostatic acid phosphatase (PAP), may also serve as antigen targets for T cells. However, TAA-based cancer vaccines have failed to produce satisfactory results in clinical trials [25, 26]. The first cancer vaccine approved by Food and Drug Administration (FDA), Sipuleucel-T, used PAP protein as the antigen target along with other immunostimulatory factors. This vaccine showed only a moderate improvement in the median survival time for patients with prostate cancer [27]. Modern TAA-vaccine clinical trials combined with immunotherapy treatments, such as ICI, might show more promising clinical outcomes than monotherapy [28]. However, even if TAA expression in normal tissues is barely detectable, the immune-tolerance towards TAAs arising during immune system development may render the response magnitude elicited by TAA vaccines.

### Oncogenic virus-derived antigens

Oncogenic viruses such as Epstein–Barr virus (EBV) and human papillomavirus (HPV) have been shown to be associated with the development of a variety of cancers, including cervical, nasopharyngeal, and oral cancers. Non-human-sourced cancer-related antigens from HPV and EBV are potential alternative candidates for cancer vaccines [29–31]. Classic HPV vaccines, which depend mainly on the humoral immune response, show prophylactic effects in reducing the incidence of HPV-associated cancers. Many clinical trials have shown that therapeutic HPV vaccines derived from antigens of the high-risk HPV subtypes 16 or 18 can activate CD8^+^ T cells via a cellular immune response, thereby inducing targeted cytotoxicity to the infected cancer cells [30]. These trials suggest the feasibility of using oncogenic viral vaccines with specific non-self antigens.

### Tumor-specific antigens/neoantigens

Unlike TAAs, TSAs/neoantigens are mutated peptides derived from genetic alterations of cancer genomes that are specific expression in tumor cells and do not exist in normal tissues but may elicit an anti-cancer immune response. Owing to the restricted expression within tumor cells, neoantigen-targeted immunotherapy displays high tumor specificity and reduced off-target toxicity. A clinical study of adoptive T cell therapy showed that a patient with widely metastatic cholangiocarcinoma who progressed through multiple chemotherapy regimens showed significant tumor regression after infusion of tumor-infiltrating CD4^+^ T cells of *ex vivo* expanded, and these T cells recognized an immunogenic neoantigen derived from the mutated *ERBB2IP* (ERBB2IP E805G), which encoding the ERBB2 interacting protein and highly expressed in both the original and recurrent lung lesions. This study also demonstrated that personalized neoantigen-based immunotherapies could elicit a strong anti-cancer immune response against tumor cells. Another clinical study using neoantigen-based cancer vaccines showed that through vaccination with multiple personalized neoantigens, cancer patients could attain significant tumor regression with expansion of both the pre-existing neoantigen-specific T-cells and the repertoire of neoantigen-specific T cells *in vivo* [32]. When compared to CTA-based cancer vaccines or adoptive cell therapy, cancer vaccines with neoantigen have several advantages, including being multi-target and having a broad spectrum. Moreover, a neoantigen vaccine could induce a continuous anti-tumor immune response by generating memory T cells [16, 33, 34].

#### Neoantigen Identification

**Fig. 2:**
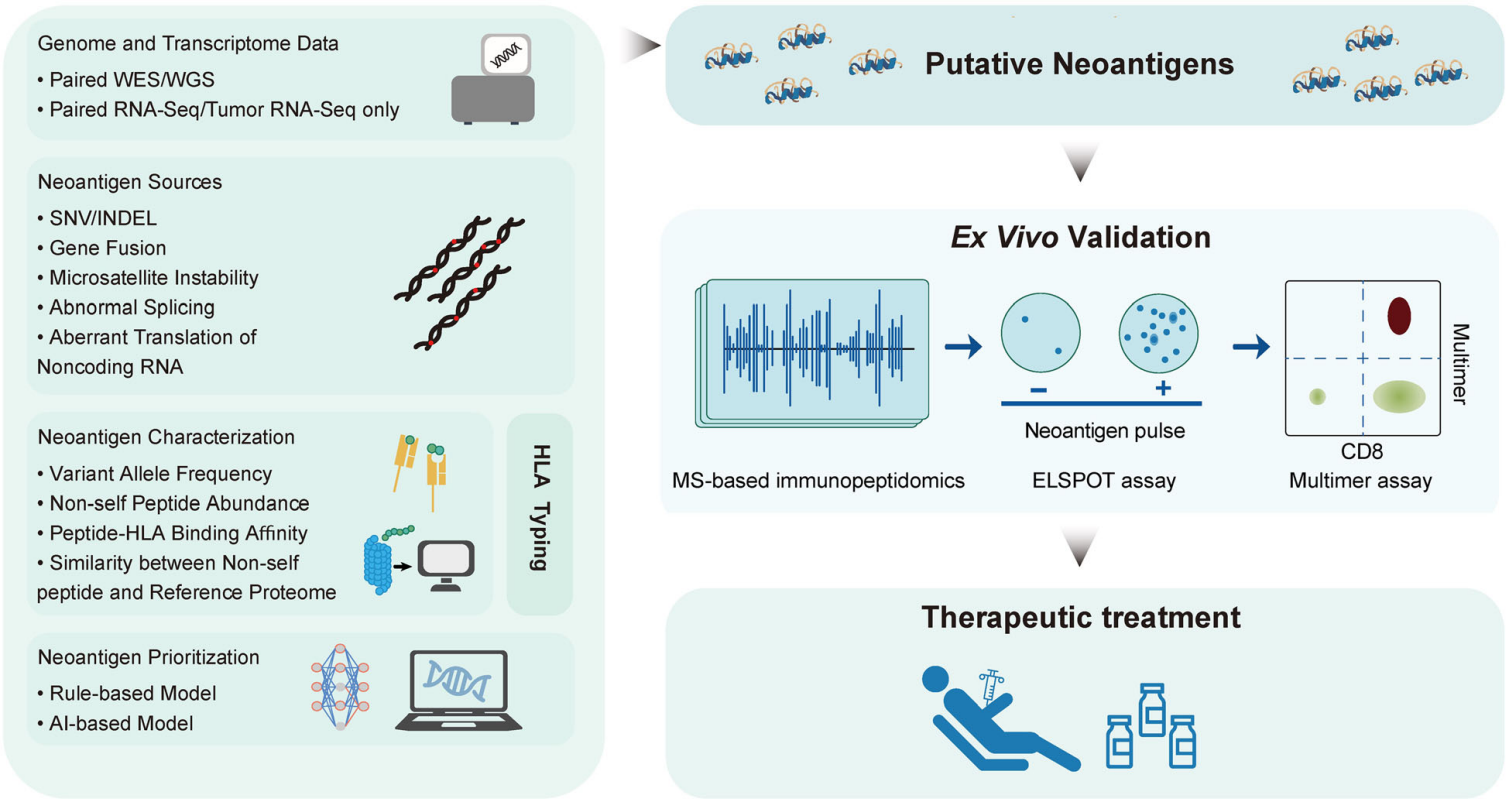
Neoantigen prediction and validation workflow. Left panel corresponds to the workflow of *in silico* neoantigen prediction. Next, putative neoantigens obtained from neoantigen prediction are subject to *ex vivo* validation where various techniques, such as mass spectrometry, Elispot and MHC Tetramer are used for selecting presented and immunogenic neoantigens for clinical applications

Neoantigen identification relies on high-throughput sequencing data derived from DNA and RNA samples from paired tumor and normal tissues. By analyzing whole exome sequencing (WES) and mRNA transcriptome sequencing (RNA-Seq) data using bioinformatics methods, mutations in the DNA and RNA levels, which could possibly result in neoantigen epitopes, can thus be determined. Typically, non-synonymous single nucleotide variants (SNVs) and DNA insertions or deletions have been the only two sources for neoantigen prediction across multiple studies [32, 35]. However, only focusing on these two types of mutations tends to underestimate potential neoantigens that tumors may display. It has been reported that neoantigens can be originated from many other types of sources, including (1) gene fusion events; (2) splice-site creation mutations (SCMs); (3) mRNA intron retention; and (4) endogenous retroelements [36]. Notably, neoantigens derived from other types of mutations such as gene fusion and SCM, tend to be more immunogenic than SNV-derived neoantigens, suggesting that incorporating more sources of neoantigens will not only increase the number of neoantigens in the vaccine but also enhance the quality and efficacy of neoantigen vaccines [37, 38]. Next, the identified mutations are computationally translated to mutated peptides (typically 8 to 15 amino acids in length for class I HLAs and 13 to 25 amino acids in length for class II HLAs) and filtered for neo-peptides that are distinct from any wildtype sequence within the human proteome. Finally, multiple measurements related to neoantigen expression, presentation, and recognition were calculated to build an integrative model for neoantigen prediction. A recent survey study revealed that significant measurements associated with neoantigen immunogenicity include (1) binding affinity between the mutated peptide and HLA alleles of the patient being studied; (2) binding stability of the peptide-HLA complex, and (3) expression of the host gene [39]. In addition to features derived from WES and RNA-Seq data, other features depicting recognition potential between mutated peptides and T cell receptors can also be incorporated into the neoantigen prediction system [40] (Fig. 2).

#### Neoantigen-based therapies

Neoantigen-based tumor therapies mainly include synthetic long peptide (SLP) vaccines, nucleic acid (DNA/mRNA) vaccines, dendritic cell (DC)-based vaccines, neoantigen-specific TCR-T cell-based therapies, and bispecific antibodies associated with public neoantigens (Fig. 1b). Their characteristics are summarized in Table 2. Clinical trials of these personalized neoantigen-based therapies have been conducted in patients with advanced-stage cancers globally.

**Table 2:** Characteristics of neoantigen-based therapy

Therapy type	Peptide vaccine	DNA vaccine	mRNA vaccine	Dendritic cell (DC) vaccine	T cell-based therapy
Material	Synthetic long peptide	Double stranded (dsDNA)	mRNA encapsulation in carriers	Neoantigen-loaded autologous dendritic cells	Neoantigen-specific autologous T cell
Immunogenicity	Low/Moderate	Low/Moderate	Low/Moderate	High	High
Neoantigen number	Up to 20 ~ 30	Up to 20 ~ 30	Up to 20 ~ 30	Up to 20 ~ 30	Few
Human leucocyte (HLA)-subtype restricted	Yes	No	No	Yes	Yes
Advantage	Low toxicity; easy to manufacture	Low toxicity; easy to manufacture on large scale	Low toxicity; easy to manufacture on large scale	N/A	N/A
Difficulty	Restricted to HLA subtype; costly and difficult purification of proteins and equipping the proteins with natural, post-translational modifications.	Need to enter the nucleus to express the antigen	Improving mRNA stability and preventing from degradation; harsh storage condition	Expensive; labor-consuming DC generation	Expensive; labor-consuming identification and isolation of these mutation specific tumor-infiltrating lymphocytes (TILs) and T cell receptors (TCRs)
Severe side effect	N/A	N/A	N/A	N/A	Cytokine release syndrome

### Synthetic long-peptide (SLP) vaccines

When using peptide vaccines, it is important to choose short immunogenic sequence (8 to 10-mer) or long sequence (25-mer), the latter of which requires an additional processing step to chop them into immunogenic peptides. It could overcome the immune tolerance and successfully induce CD4^+^ and CD8^+^ T cell responses by extending short peptides into long peptides [41]. Following injection of the peptide-based vaccines into patients via the subcutaneous route, these peptides are taken up by antigen-presenting cells (APCs) around the injection sites. Through endocytosis and lysosomal processing, these APCs mature and load antigen epitopes onto MHC/HLA molecules. The mature APCs, loaded with MHC-I or MHC-II molecules, migrate to adjacent draining lymph nodes where they present neoantigens and activate CD8^+^ cytotoxic T cells or CD4^+^ T helper cells, respectively. Activation of T cells requires a primary signal from the MHC/HLA–Ag–TCR tertiary complex and secondary signals based on both cell–cell interactions and secreted immunomodulators such as IFN-γ [42]. Next, activated T cells undergo clonal expansion, exit the lymph nodes into the circulatory system, and migrate to tumor sites, where they recognize tumor cells with cognate neoantigens present on their surface and conduct cell killing. Although peptide-based neoantigen vaccines have been employed in several clinical trials, the application of neoantigen peptides faces challenges with respect to rapid and affordable manufacturing, the lack of natural post-translational modification, and difficulty in synthesis and purification due to variations in length, charge, and hydrophobicity.

Poly-ICLC is a synthetic double stranded RNA (dsRNA) mimetic that stimulates both TLR3 and MDA5. By stimulating MDA5, Poly-ICLC potently induces IFN-I and IL-15. It can also promote T-cell expansion and enhance T-cell infiltration, making it a potent adjuvant for peptide cancer vaccines [43]. In recent years, several major clinical trials have tested the efficacy of peptide cancer vaccines. Ott et al. conducted a clinical trial of NeoVax, an individualized neoantigen-based peptide vaccine for patients with melanoma (NCT01970358). Patients were pathologically confirmed as high-risk melanoma patients with stage IIIB/C and IVM1a/b and were treated with NeoVax after a median of 18 weeks postoperatively. Individualized peptide vaccines targeting up to 20 neoantigens were formulated with Poly-ICLC. The results showed that this treatment was safe and immunogenic [32].

In 2021, Ott et al. published the results of a follow-up of eight melanoma patients who had participated in this trial, with a median follow-up time of 55 months. The results showed that all eight patients were alive, and six patients showed no signs of active disease. Testing of T cells in the patients’ peripheral blood revealed that neoantigen-specific T cell responses persisted in melanoma patients for several years after NeoVax vaccination and that neoantigen-specific T cells exhibited a memory phenotype [44]. Notably, in another trial of neoantigen-specific peptide vaccines for newly diagnosed glioblastoma conducted by Ott et al., the results demonstrated that peripheral blood neoantigen-specific T cells could migrate into an intracranial glioblastoma tumor [45].

Theoretically, lysis of tumor cells would release the new tumor neoantigens or TAAs, which could trigger new anti-tumor immune responses. The scientists subsequently also demonstrated that NeoVax induces epitope spreading of T-cell responses, indicating tumor cell lysis, which also implies broadening of the spectrum of tumor-specific cytotoxicity. However, three patients in this study had relapsed at 26, 40 and 40 months after vaccination, and two others had relapsed soon after vaccination, but they achieved complete remission soon after anti-PD-1 pembrolizumab treatment [44].

Immune checkpoint inhibitors have revolutionized the treatment of cancer patients over the past decade. Anti-PD-1/PD-L1 antibodies, based on the principle of unlocking immunosuppressive signaling have shown significant antitumor activity in multiple tumor types [46]. It is natural to consider a combination of ICI and other anti-cancer therapies to determine whether the combination is better than monotherapy for cancer patients. A phase Ib trial (NCT02897765) investigated the efficacy of a personalized neoantigen vaccine, NEO-PV-01, combined with PD-1 blockade in treatment of advanced melanoma, non-small cell lung cancer, or bladder cancer patients. In this trial, after *in silico* neoantigen prediction and selection for patients with high tumor mutational burden, corresponding good manufacturing practice grade neoantigen peptides were generated, then mixed with adjuvant poly-ICLC and administered subcutaneously. Nivolumab was administered during the post-vaccine, vaccine and post-vaccine periods. The results showed that the overall response rate was 59 %, 39 and 27 % for the melanoma, non-small cell lung cancer and bladder cancer patients, respectively; median progression-free survival (PFS) was 23.5 months, 8.5 and 5.8 months, respectively; and the 1-year overall survival was 96 %, 83 and 67 %, respectively. These data are not inferior to the historical data of PD-1 antibody monotherapy. Epitope spreading was also observed after vaccination, and was associated with longer PFS [47].

### Nucleic acid (DNA/mRNA) vaccines

It has become a versatile technology to use nucleic acid-based vaccines to deliver DNA or mRNA encoding targeted epitopes for the prevention of infectious diseases and cancer treatment [48, 49]. DNA/mRNA-based neoantigen vaccines function via a similar processing step to peptide-based vaccines, except for an additional translation and/or transcription step(s) in DCs [49, 50]. Nucleic acid-based vaccines have been delivered in various formats, including encapsulation by delivery carriers, such as lipid nanoparticles (LNPs), which were prepared by ethanol injection nanoprecipitation by mixing acidified RNA and lipids dissolved in ethanol [50]. Compared to peptides, nucleic acid formulations facilitate the continuous and effective expression of antigens and immune stimulation. Importantly, nucleic acid formulations produce antigen peptides from within the cell, avoiding the costly and difficult purification of proteins as well as equipping the proteins with natural, post-translational modifications. Nucleic acid vaccines provide advantages in efficacy, shortened design and manufacturing time, as well as production scalability and reliability [50]. Comparing the two, DNA carries genetic information and need to enter the nucleus to express the antigen, while mRNA directs antigen production in a better and focused manner without entering the nucleus. A further clear advantage of mRNA vaccines compared with DNA vaccines is the expression in non-dividing cells is relatively high, and there is no risk of integration into the host genome. In addition, given its intrinsic immunogenic features, mRNA could function as an adjuvant and lower doses are required to elicit an optimal immune response, making it a safe and promising platform for neoantigen vaccines. Currently neoantigen-targeted DNA/mRNA cancer vaccines have also been tested in various clinical trials.

A personalized RNA-lipoplex neoantigen-based vaccine RO7198457, encoding up to 20 neoantigens based on neoantigen prediction, was tested in previously heavily treated patients with advanced-stage solid tumors in a phase 1b trial (NCT03289962). In total, 29 patients received RO7198457 as monotherapy at escalated dosages, and 132 patients received RO7198457 combined with the anti-PD-L1 antibody atezolizumab. The most common tumor types in this study were non-small cell lung cancer, colorectal cancer, melanoma, and breast cancer; the majority of patients had low levels of PD-L1 expression. Based on data from patients who underwent at least one tumor assessment, the trial showed an objective response rate of 4 % (1/26) and a stable disease rate of 40 % (9/26) in the monotherapy cohort and an objective response rate of 8 % (9/108) and a stable disease rate of 49 % (53/108) in the combination cohort. RO7198457 induced neoantigen-specific T cell responses in the majority of patients in both groups.

Another mRNA lipid-encapsulated RNA-based neoantigen-based vaccine mRNA-4157 was tested in a phase 1 trial (NCT03313778). Among 79 patients treated with mRNA-4157, 16 were treated as monotherapy and 63 in combination with the immune checkpoint inhibitor pembrolizumab. mRNA-4157 was safe and well tolerated. Three complete remissions (CRs) (one head and neck squamous cell carcinoma (HNSCC), one microsatellite instability-high (MSI-H) colorectal cancer, and one MSI-H prostate cancer) and eight partial remissions (PRs) (one bladder cancer, four HNSCC, two small cell lung cancer, and one MSI-H endometrial cancer) were observed in the combination group. Particularly in the 10 CPI-naive HPV-negative HNSCC patients, the response rate was 50 % (1 CR, 4 PR, 4 stable disease (SD)) and mPFS was 9.8 months, which compared favorably with the published response rate of approximately 14.6 % and mPFS of 2.0 months for pembrolizumab monotherapy [51].

### DC vaccines and neoantigen-specific TCR-T cell-based therapy

As an alternative, DC vaccines [52], which contain autologous DCs isolated from the peripheral blood of a patient, are loaded with neoantigen peptides *ex vivo* before reinfusion to the body, thereby bypassing the processes of antigen capture, processing, presenting, and DC maturation *in vivo* [53].

Recently, the results of a phase I trial (NCT02956551) were revealed. It investigated the safety and efficacy of a personalized neoantigen peptide-pulsed autologous DC vaccine (Neo-DCVac) for 12 heavily treated metastatic lung cancer. The vaccine was safe and showed an objective effectiveness rate of 25 %, a disease control rate of 75 %, a mPFS of 5.5 months, and a median overall survival (mOS) of 7.9 months. When used in combination with ICI treatment, Neo-DCVac showed synergistic therapeutic effects in four patients receiving disease control (two PRs, two SDs) who previously showed no primary response to or relapse from ICI treatment. These results revealed that Neo-DCVac could induce specific T cell immunity and therapeutic benefits [54]. This trial provided the first evidence of the efficacy of a neoantigen-based DC vaccine in cancer patients.

TCR-engineered T cell-based therapy is another option for neoantigen application, which requires genetically engineered T-cells from patients with receptors that can recognize their own tumor-specific neoantigens. Compared to DC vaccine trials, there are no available results of clinical trials for neoantigen-specific TCR-T cell-based therapy, and only case reports have been published.

In addition to the previously discussed case report in which the tumor of the cholangiocarcinoma patient harbored a neoantigen ERBB2IP E805G, Rosenberg reported several cases treated in a similar manner, including a patient with metastatic colorectal cancer infused with KRAS-G12D targeted TILs and a chemorefractory HR-positive patient with metastatic breast cancer treated with TILs against four mutations distributed in SLC3A2, KIAA0368, CADPS2, and CTSB, respectively. The additional two cases both achieved clinical response, and the patient with breast cancer who was treated with T cell therapy in combination with interleukin-2 and ICI showed complete durable regression for more than 22 months while the patient with metastatic colorectal cancer who only received infusion with KRAS-G12D targeted TILs progressed 9 months after therapy [55, 56].

However, one of the biggest challenges for neoantigen-based T-cell therapies is the labor-intensive process of identification and isolation of these mutation-specific TILs and TCRs. Regarding neoantigen-based DC and TCR-T therapy, it is noteworthy that the infusion of immune cells into the human body can cause severe and sometimes fatal side effects. Cytokine release syndrome (CRS) is the most frequent of these, which causes the activation in the body’s immune cells to release large amounts of cytokines [57].

### Cancer vaccines based on public neoantigens

Compared with purely personalized neoantigens, public or shared neoantigens are derived from driver mutations in oncogenes or other hotspot mutations across the genome. They are characterized as immunogenic epitopes presented in a subset of patients with a given cancer subtype. Therefore, the discovery of public neoantigens is dependent on the analysis of individualized neoantigens from a patient pool of considerable size. Once identified and evaluated for their immunogenicity *ex vivo*, public neoantigens could be manufactured in advance and become readily off-the-shelf for patients upon genetic testing. One major advantage of the public neoantigens is that they can be quickly administered to cancer patients, particularly for those with late-stage cancers and those with only a short therapeutic window for treatment. In addition, a cancer vaccine with public neoantigens would lower the cost of treatment.

To date, some studies have attempted to identify public neoantigens as well as TCRs associated with public neoantigens. One example of a public neoantigen corresponds to the mutation of G12D on KRAS, which is frequently found in pancreatic adenocarcinoma, colon adenocarcinoma, non-small cell lung, and colorectal cancer [33]. The case report of metastatic colorectal cancer patients discussed previously showed a primary response to KRAS-G12D targeted TILs. Similarly, TP53, a well-known tumor suppressor gene widely mutated in a large number of cancers, has a broad range of hotspot mutations and is shared by multiple cancers [58]. Malekzadeh et al. developed a TP53-specific screening assay to assess T cell responses to neoepitopes associated with TP53 hotspot mutations. As a result, antigen-specific T cells were observed in common across a significant portion of patients [59].

In contrast, Okada et al. identified a TCR that can recognize a synthetic peptide encompassing the H3.3K27M mutation presented by HLA-A2, which is a driver mutation and diagnostic biomarker of diffuse midline glioma. T cells transduced with TCR showed a significant ability to suppress tumor progression of glioma xenografts in mice [60]. Similarly, van der Lee et al. identified a TCR that can recognize an NPM1 hotspot frameshift mutation that occurs in ~ 30 % AML. Preclinical experiments confirmed the anti-tumor response of T cells transduced with this TCR [61].

Currently, around 40 clinical studies employing public cancer neoantigens can be tracked from ClinicalTrials.gov. Most of these public neoantigen clinical trials are still ongoing, while some of the early trials have reported favorable outcomes. High safety and immunogenicity were observed in an open-label, single-armed phase I clinical trial (NCT01250470), in which nine recurrent-malignant-glioma patients were vaccinated with a 15-amino acid-long survivin (also known as baculoviral inhibitor of apoptosis repeat-containing 5, BIRC5) peptide, containing different eight-to-ten-amino acid immunoreactive epitopes with the same C57M mutation [62]. This mutated peptide vaccine was able to induce a humoral immune response and certain HLA allele-restricted T cell responses *in vivo*. Some patients presented with a partial response or stable disease for at least six months. Moreover, another study investigating the role of survivin in neuroendocrine neoplasms revealed that ionizing radiation could induce survivin expression in human carcinoid cell lines [63].

In another trial with a similar design (NCT02261714), low or high doses of the KRAS mutation peptide TG01, consisting of seven well-known oncogenic mutations in codons G12 and G13, were used as vaccines, co-administrated with granulocyte-macrophage colony-stimulating factor (GM-CSF) in order to enhance T cell response, in 32 stage I or II pancreatic adenocarcinoma patients who had undergone surgical resection (R0 or R1) and of whom 93.75 % harbored a detectable KRAS mutation. The results showed that a lower dose resulted in good safety outcomes, whereas a few severe adverse reactions were observed in the higher-dose group, possibly related to the treatment. Both doses produced strong cellular immunological responses, and subject survival rates at two and three years were approximately 72 and 37 %, respectively [64]. Taken together, public neoantigen vaccines have proven their great value as therapeutic targets in cancer treatment.

### Bispecific antibodies associated with public neoantigens

Carcinogenesis can be driven by inactivating mutations in tumor suppressor genes, such as TP53 and APC. However, the protein products of these mutated driver genes are usually incomplete, biologically inactive and intracellularly degradable, which makes it difficult to develop antibodies and small molecule drugs to target these proteins and develop a novel form of neoantigen-based therapy.

Bispecific antibodies are a class of synthetic antibodies that can simultaneously target two antigens, this bridges the effector and target cells and provides better synergistic effects [65]. After binding to antigens, bispecific antibodies can induce specific activation of effector cells in the presence of target cells, such as T cells and tumor cells. Recently, a preclinical study using bispecific antibodies targeting a neoantigen derived from the hotspot mutation R175H in the TP53 gene has provided new insights into neoantigen-based cancer therapies. Hsiue et al. identified TCR-mimic single-chain variable fragments (scFvs) specific for a p53 R175H peptide from scFv-expressing phage clones, which are HLA-A*02:01-restricted. The scFvs were converted to T cell-retargeting bispecific antibodies by linking each individual scFv to an anti-CD3e scFv (UCHT1) in a single-chain diabody (scDb) format [66]. Finally, researchers identified a diabody clone named H2-scDb that could bind to p53^R175H^/HLA-A*02:01 at low concentrations. The anti-tumor immunity of the diabody were then validated by activating T cells to secrete cytokines and suppressing the growth of human xenograft tumors in mice. Almost simultaneously, the same group preclinically explored the effects of bispecific antibodies targeting RAS-derived neoantigens in a similar manner. As a result, the bispecific antibodies targeting neoantigens derived from G12V (presented by HLA-A3) and Q61H/L/R (presented by HLA-A1) also showed potent anti-tumor immunity *in vitro* and *in vivo* [67]. The success of bispecific antibodies targeting public neoantigens derived from proto-oncogenes and tumor suppressor genes demonstrates the potential of this novel neoantigen-based therapy in treating cancers bearing previously defined undruggable mutations.

## Conclusion and perspectives

Precision medicine, particularly personalized or public neoantigen vaccine, represents cutting-edge advances and prospects for cancer treatment. Derived from multiple and variable sources, neoantigens have been proven to be tumor-specific and highly immunogenic, and they can generate long-term memory for immune protection against cancer. Neoantigen-based antitumor therapies have made tremendous progress in recent years, both in terms of identification, prediction, or screening of neoantigens and therapeutic options [68]. Clinical trials using neoantigen vaccines have reported high safety and efficacy in multiple cancer subtypes, and many additional trials are ongoing [32, 35, 45, 69]. As a new and promising technology, neoantigen vaccines using mRNA as a delivery formulation and public neoantigens as targets show improved clinical properties and druggability, and have attracted considerable interest for the development of next-generation precision cancer immunotherapy. However, some challenges remain, with several aspects of neoantigen vaccines yet to be optimized, to achieve better clinical responses.

The time taken for neoantigen identification and manufacturing is relatively long, which requires at least 6–8 weeks and does not leave enough time for patients with a short window of treatment. Given the low accuracy of the currently available neoantigen prediction algorithms, considerable effort will be required to utilize the machine-learning platform to improve neoantigen prediction accuracy.

Additionally, optimal neoantigen formulation and corresponding modifications, the utilized neoantigen delivery system and routes, and the safe and efficient administration dosages need to be tested and evaluated in ongoing and future clinical trials. Moreover, neoantigen vaccines combined with TAAs and other immunomodulator therapies have shown improved therapeutic effects. A study by Ott et al. demonstrated that neoantigen-based vaccines would release the new tumor neoantigens or TAAs, which could trigger additional anti-tumor immune responses. When used in combination with ICIs, synthetic long peptide vaccines such as NeoVax and NEO-PV-01; mRNA vaccines such as RO7198457 and mRNA-4157; DC vaccines such as Neo-DCVac; and neoantigen-based T cell therapies, such as KRAS-G12D-targeted TILs, all of them showed synergistic therapeutic effects. In addition to combining ICIs with neoantigen vaccines to reduce immunosuppression in the tumor microenvironment, another direction for boosting the performance of neoantigen vaccines is to combine them with radiotherapy. Multiple lines of evidence have shown that radiotherapy can upregulate the expression of host genes of highly immunogenic neoantigens with a low abundance of the HLA-epitope complex [70, 71]. Therefore, combination therapy using neoantigen vaccine along with radiotherapy, chemotherapy, or ICIs and TME immunomodulators should be further evaluated in future studies.

### Availability of data and materials

The material supporting the conclusions of this review are included within the article.

## Data Availability

The material supporting the conclusions of this review are included within the article.

## Abbreviations

ICI/ICB/ICP/CPI: Immune checkpoint inhibitors
PD-1: Programmed cell death protein-1
PD-L1: Programmed cell death ligand-1
CTLA-4: Cytotoxic T lymphocyte antigen-4
ACT: Adoptive cell transfer
HLA: Human leukocyte antigen
TILs: Tumor-infiltrating lymphocytes
TCR: T-cell receptor
Ag: Antigen
MHC: Major histocompatibility complex
TAAs: Tumor-associated antigens
TSAs: Tumor-specific antigens
HER2: Human epidermal growth factor receptor 2
TERT: Telomerase reverse transcriptase
TYR: Tyrosinase
CTAs: Cancer testis antigens
CDAs: Cell lineage differentiation antigens
NY-ESO-1: New York esophageal squamous cell carcinoma-1
MAGE: Melanoma antigen gene
CEAs: Carcinoembryonic antigens
PSA: Prostate-specific antigen
PAP: Prostatic acid phosphatase
FDA: Food and Drug Administration
HPV: Human papillomavirus
EBV: Epstein–Barr virus
WES: Whole exome sequencing
RNA-Seq: mRNA transcriptome sequencing
SNV: Single nucleotide variant
SCMs: Splice-site creation mutations
SLP: Synthetic long peptide
DC: Dendritic cell
APCs: Antigen-presenting cells
PFS: Progression free survival
LNP: Lipid nanoparticles
CR: Complete remission
HNSCC: Headneck Squamous Cell Carcinoma
MSI-H: Microsatellite instability-high
PR: Partial remission
SD: Stable disease
CRS: Cytokine release syndrome
GM-CSF: Granulocyte-macrophage colony-stimulating factor
scFv: Single-chain variable fragment
scDb: Single-chain diabody
